# Epithelial EP4 plays an essential role in maintaining homeostasis in colon

**DOI:** 10.1038/s41598-019-51639-2

**Published:** 2019-10-23

**Authors:** Yoshihide Matsumoto, Yuki Nakanishi, Takuto Yoshioka, Yuichi Yamaga, Tomonori Masuda, Yuichi Fukunaga, Makoto Sono, Takaaki Yoshikawa, Munemasa Nagao, Osamu Araki, Satoshi Ogawa, Norihiro Goto, Yukiko Hiramatsu, Richard M. Breyer, Akihisa Fukuda, Hiroshi Seno

**Affiliations:** 1 Department of Gastroenterology and Hepatology, Kyoto University Graduate School of Medicine, Kyoto, Japan; 2 Department of Drug Discovery Medicine, Medical Innovation Center, Kyoto University Graduate School of Medicine, Kyoto, Japan; 3 Sumitomo Dainippon Pharma Co., Ltd, Osaka, Japan; 4 Department of Medicine, Vanderbilt University, Nashville, TN 37232 USA

**Keywords:** Ulcerative colitis, Experimental models of disease, Chronic inflammation

## Abstract

Colonic epithelial cells comprise the mucosal barrier, and their dysfunction promotes microbial invasion from the gut lumen and induces the development of intestinal inflammation. The EP4 receptor is known to mediate the protective effect of prostaglandin (PG) E_2_ in the gastrointestinal tract; however, the exact role of epithelial EP4 in intestinal pathophysiology remains unknown. In the present study, we aimed to investigate the role of epithelial EP4 in maintaining colonic homeostasis by characterizing the intestinal epithelial cell-specific *EP4* knockout (*EP4* cKO) mice. Mice harboring the epithelial EP4 deletion showed significantly lower colonic crypt depth and lower numbers of secretory cell lineages, as well as impaired epithelial cells in the colon. Interestingly, EP4-deficient colon epithelia showed a higher number of apoptotic cells. Consistent with the defect in mucosal barrier function of colonic epithelia and secretory cell lineages, *EP4* cKO colon stroma showed enhanced immune cell infiltration, which was accompanied by increased production of inflammatory cytokines. Furthermore, EP4-deficient colons were susceptible to dextran sulfate sodium (DSS)-induced colitis. Our study is the first to demonstrate that epithelial EP4 loss resulted in potential “inflammatory” status under physiological conditions. These findings provided insights into the crucial role of epithelial PGE_2_/EP4 axis in maintaining intestinal homeostasis.

## Introduction

Intestinal homeostasis is regulated by the cross-talk between epithelial cells and stromal cells, including immune cells, fibroblasts, and vascular cells, which occurs concomitantly with the interactions between dietary nutrients and microorganisms. Prostaglandin (PG) E_2_ is one of the key mediators in complicated cross-talk in intestinal homeostasis. PGE_2_ is synthesized by cyclooxygenase 1 and 2 and acts by binding to four PGE receptors (EP1, EP2, EP3, and EP4). Among these EP receptors, EP4 is known to be expressed in both colonic epithelial cells^1^ and in lamina propria cells, such as TH17 cells^2^ and naive CD4^+^ T cells^3^.

PGE_2_ plays a pivotal role in colonic pathophysiology, such as inflammation and cancer^4–8^. Indeed, multiple researchers reported the protective roles of PGE_2_ and EP4 in mouse experimental colitis or human inflammatory bowel diseases (IBDs)^9,10^. For example, EP4 global knockout (KO) mice showed the more severe dextran sulfate sodium (DSS)-induced colitis relative to mice deficient for other EP receptor subtypes^9^. Administration with EP4 agonists was reported to ameliorate colitis in mouse experimental models by inhibiting the production of inflammatory cytokines from infiltrating immune cells^9^. Results of a phase 2 clinical trial of an EP4-selective agonist demonstrated its potential beneficial effect for ulcerative colitis patients^10^. However, while PGE_2_/EP4 signaling is known to affect many cell types, including both epithelial and stromal cells, to regulate the immune system in a pleiotropic manner, these previous studies have not clarified the role of epithelial cell-specific function of EP4 for the maintenance of colonic homeostasis under physiological conditions. Whether epithelial EP4 itself is involved in the repression of the intestinal inflammation, such as IBD, has not yet been explored. Here, we examined the colonic mucosa of epithelial cell-specific *EP4* knockout (*EP4* cKO) mice and showed that the lack of epithelial EP4 leads to alterations in intestinal crypt architecture and causes the inflammatory phenotype under both physiological and pathological conditions.

## Results

### Epithelial-specific deletion of EP4 impairs colon homeostasis

**Figure 1:**
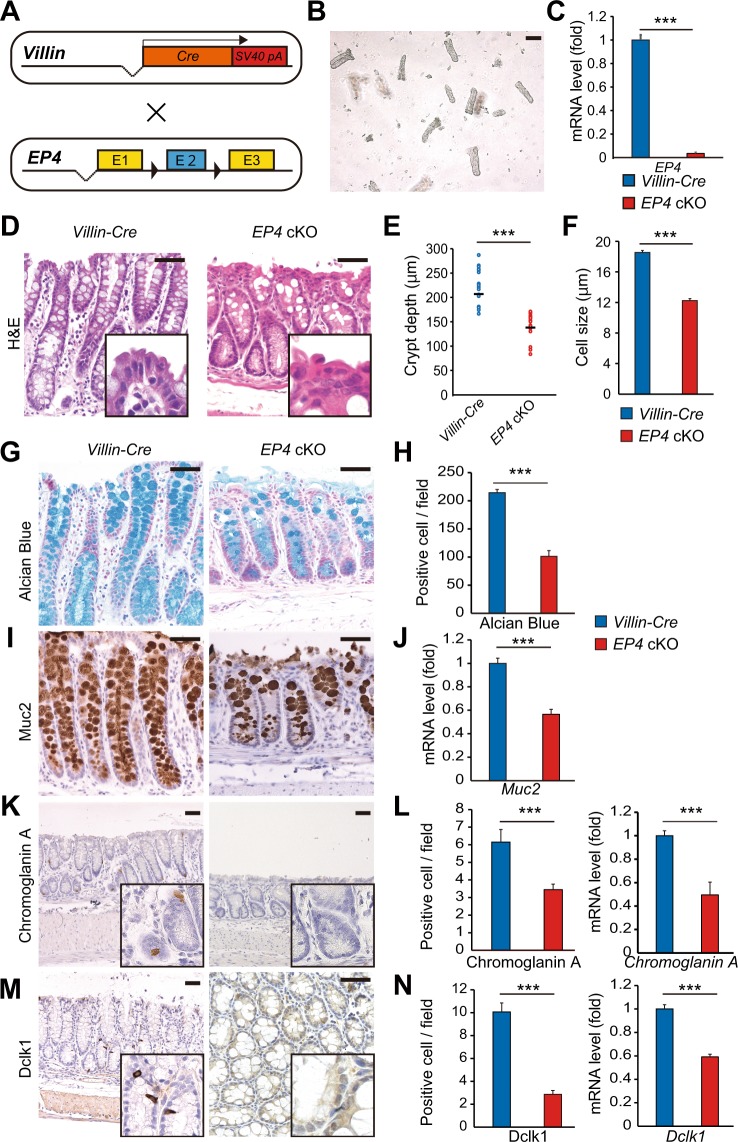
Colon homeostasis is impaired in epithelial EP4-deficient mouse. (**A**) Schema of recombination in *Villin-Cre; EP4*^*flox/flox*^ mice. (**B**) Representative microscopic view of isolated crypts. Scale bars = 100 μm. (**C**) qRT-PCR analysis of *EP4* mRNA levels in *Villin-Cre* (n = 5) and *EP4* cKO (n = 5) colon crypts from the mice at 8 weeks of age. (**D**) Hematoxylin and eosin (**H**,**E**) staining of colon for *Villin-Cre* and *EP4* cKO mice. Scale bars = 50 μm. (**E**) Crypt length in *Villin-Cre* and *EP4* cKO mouse colons (n = 4). (**F**) Cell size of colonic epithelial cells located in the top of colon crypts in *Villin-Cre* and *EP4* cKO mice (n = 3). (**G**,**H**) Alcian Blue staining and quantification in the colons from *Villin-Cre* and *EP4* cKO mice at 8 weeks of age (n = 3). Scale bars = 50 μm. (**I**) Muc2 staining of colons in *Villin-Cre* and *EP4* cKO mice at 8 weeks of age. Scale bars = 50 μm. (**J**) qRT-PCR analysis of *Muc2* mRNA levels in *Villin-Cre* and *EP4* cKO mice (n = 5). (**K**) Chromogranin A staining of colon in *Villin-Cre* and *EP4* cKO mice at 8 weeks of age. Scale bars = 50 μm. (**L**) Quantification of (**K**) (n = 3) and mRNA expression levels of *Chromogranin A* in *Villin-Cre* and *EP4* cKO mice (n = 4) analyzed by qRT-PCR on colon crypts. (**M**) Dclk1 staining of colon in *Villin-Cre* and *EP4* cKO mice at 8 weeks of age. Scale bars = 50 μm. (**N**) Quantification of (**M**) (n = 3) and mRNA expression levels of *Dclk1* in *Villin-Cre* and *EP4* cKO colon crypts (n = 4) analyzed by qRT-PCR. Results are shown as mean ± SEM. ***P < 0.005.

Consistent with the previous reports, nearly ubiquitous expression of EP4 was observed throughout both small intestine and colon with enrichment on surface tips^1,11^ (Supplementary Fig. S1A). To elucidate the role of epithelial EP4 in maintaining homeostasis of the colonic epithelium, we crossed *Villin-Cre* and *EP4*^*flox/flox*^ mice to generate *Villin-Cre; EP4*^*flox/flox*^ mice (*EP4* cKO) (Fig. 1A)^12^. Colonic epithelia were isolated from *EP4* cKO mice, and EP4 deletion was confirmed by qRT-PCR (Fig. 1B,C). *EP4* cKO mice were fertile, and their lifespans were comparable to control *Villin-Cre* mice. In addition, *EP4* cKO mice were macroscopically indistinguishable from *Villin-Cre* mice in terms of body weight and colon length (Supplementary Fig. S1B–D). However, in histology, the depths of crypts in the distal colon were significantly lower in *EP4* cKO mice than those in control *Villin-Cre* mice (Fig. 1D,E). Furthermore, the surface epithelial cells located at the top of crypts were smaller, irregular, and more disorganized in *EP4* cKO mice compared to those in control mice (Fig. 1D,F). PGE_2_ coupled to EP4 receptors stimulates cAMP-dependent mucin exocytosis in the colon^13,14^. Therefore, we next examined the effect of epithelial EP4 deletion on secretory cell lineages in the colonic epithelium. Interestingly, results of Alcian Blue staining showed that the number of goblet cells was approximately 50% lower in *EP4* cKO mice relative to that in control mice (Fig. 1G,H). *EP4* cKO mice were found to have a significantly lower number of colonic epithelial cells expressing *Muc2* (Fig. 1I,J). In addition, the numbers of enteroendocrine and tuft cells were significantly lower in *EP4* cKO mice based on immunohistochemistry results for chromogranin A and Dclk1, respectively (Fig. 1K–N). Consistent with the above results, qRT-PCR analyses indicated downregulated expression levels of *Chromogranin A* and *Dclk1* in *EP4* cKO colons (Fig. 1L,N). Collectively, these phenotypes suggested the crucial role of epithelial EP4 in maintaining crypt structure and secretory cell lineages in the colon.

### EP4 deficiency increases colonic epithelial cell death

**Figure 2:**
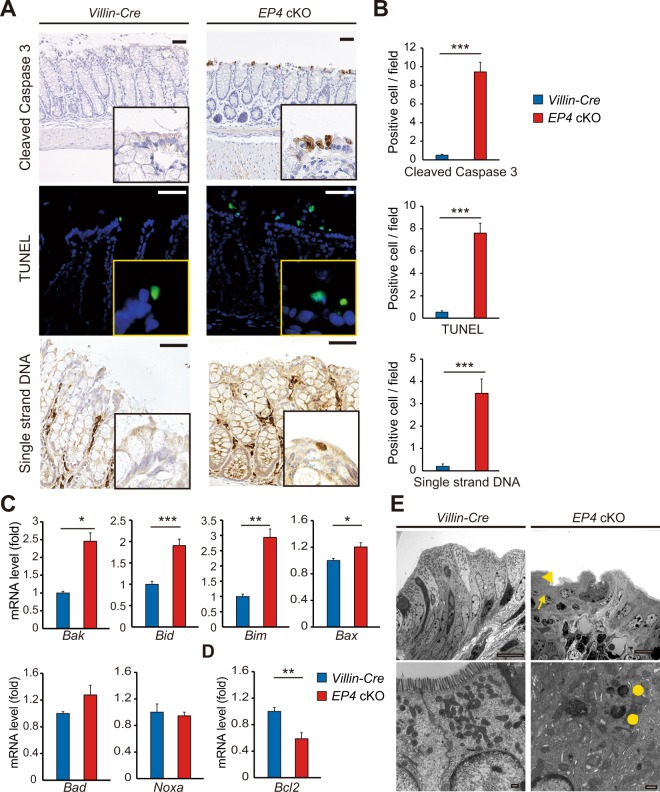
Increased apoptosis in *EP4* cKO colons. (**A**,**B**) Staining (**A**) and quantification (**B**) of Cleaved Caspase 3 (top), TUNEL (middle), and Single strand DNA staining (bottom) in *Villin-Cre* and *EP4* cKO colons (n = 3). Scale bars = 50 μm. (**C**) mRNA expression levels of apoptotic markers in *Villin-Cre* and *EP4* cKO colon crypts (n = 4) analyzed by qRT-PCR. (**D**) mRNA expression levels of an anti-apoptotic factor *Bcl-2* in *Villin-Cre* and *EP4* colon crypts (n = 4) analyzed by qRT-PCR. (**E**) Representative electron microscopic views of surface epithelial cells in *Villin-Cre* and *EP4* cKO colons. Irregular apical cell surface (arrowhead), abnormal cell polarity, fewer mitochondria (arrow), and denatured mitochondria (circles) in *EP4* cKO colons. Scale bars = 10 μm (top), 500 nm (bottom). Results are shown as mean ± SEM. *P < 0.05, **P < 0.01, ***P < 0.005.

To determine the underlying mechanisms by which epithelial EP4 loss impaired colonic homeostasis, we next examined apoptosis and proliferation status of *EP4* cKO colon epithelia. Interestingly, immunohistochemistry for cleaved caspase 3 demonstrated an increase in the number of apoptotic cells, especially on the luminal surface of the colonic epithelium in *EP4* cKO mice (Fig. 2A,B). Furthermore, results of TUNEL staining and single-stranded DNA staining showed a marked increase in the number of apoptotic cell as in the case of cleaved caspase 3 (Fig. 2A,B). Importantly, mRNA expression levels of apoptosis-associated genes, including *Bak*, *Bid*, *Bim*, and *Bax*, were significantly upregulated, whereas that of an anti-apoptotic factor *Bcl-2* was downregulated in *EP4* cKO mouse colons (Fig. 2C,D). Consistent with the observed changes in apoptosis-associated genes and their roles in a mitochondrial cytochrome c-mediated cell apoptosis pathway, electron microscopy analyses revealed fewer and denatured mitochondria and irregular apical cell surface and abnormal cell polarity in *EP4*-deficient colonic epithelia (Fig. 2E). On the other hand, Ki67 staining revealed that proliferation was not significantly altered in *EP4* cKO mouse colons (Supplementary Fig. S2A,B). Consistent with this result, we observed no significant differences in the immunostainings for β-catenin that play pivotal roles in epithelial proliferation in the intestines and the expression levels of *Myc* (encoding c-Myc) and *Ccnd1* (encoding Cyclin D1), representative target genes of Wnt/β-catenin pathway (Supplementary Fig. S2C,D). These results also keep in line with the findings that there was no significant difference between *Villin-Cre* and *EP4* cKO mice in the expression of *Lgr5* and *Sox9*, well-established markers for intestinal stem cells (ISCs) or progenitor cells and also known as Wnt/β-catenin targets (Supplementary Fig. S2D). These results indicated the importance of epithelial EP4 for crypt cell survival but not for proliferation in colonic epithelia.

**Figure 3:**
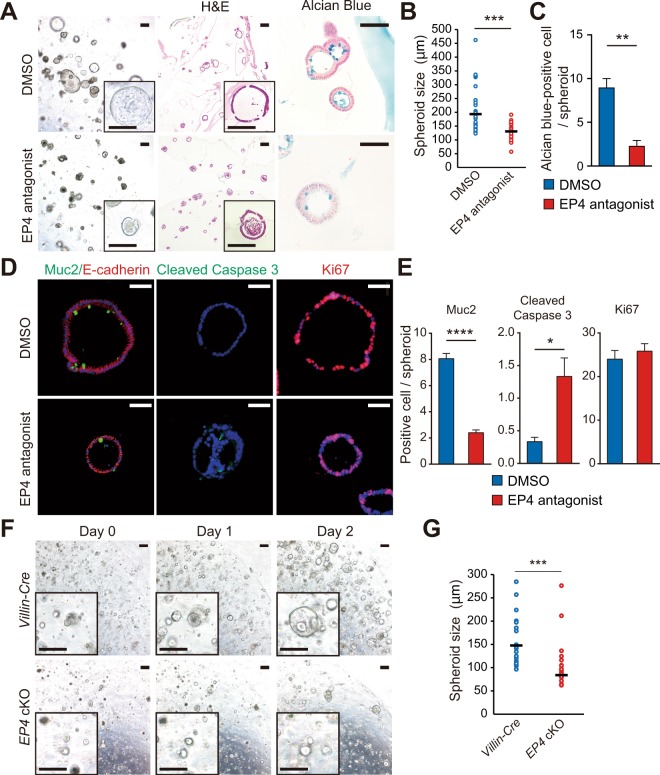
Increased apoptosis in 3D-spheroid cultures of *EP4* cKO colons. (**A**) Representative images of colon spheroids treated with or without EP4 antagonist for 48 h. 3D-culture images (left), (**H**,**E**) (middle), and Alcian blue staining (right). Scale bars = 200 μm (left, middle), 50 μm (right). (**B**) Diameter of spheroids treated with or without EP4 antagonist for 48 h (n = 29). (**C**) Quantification of Alcian blue-positive cells in (**A**) (n = 3). (**D**) Representative staining of Muc2/E-cadherin (left), Cleaved Caspase3 (middle), and Ki67 (right) on spheroids treated with or without EP4 antagonist for 48 h. Scale bars = 50 μm. (**E**) Quantification of Muc2, Cleaved Caspase 3, and Ki67 staining in (**D**) (n = 3). (**F**) Time-course images of spheroids generated from *Villin-Cre* and *EP4* cKO colon crypts. Scale bars = 200 μm. (**G**) Quantification of day 2 spheroids in (**F**) (n = 50). Results are shown as mean ± SEM. *P < 0.05, ***P < 0.005, ****P < 0.001.

To further validate the effect of EP4 deletion on epithelial cell apoptosis, we isolated WT mouse colonic epithelia and performed 3D-spheroid culture with or without administration of the EP4 antagonist, L-161,982. At 48 h after administration with the EP4 antagonist, we observed significant defects in the spheroid growth in the EP4 antagonist-treated group (Fig. 3A,B). Consistent with the findings in the *in vivo* system, the numbers of Alcian blue- (Fig. 3A,C) or Muc2-positive cells (Fig. 3D,E) representing secretory cell lineages was significantly lower in mice treated with EP4 antagonist. In addition, Cleaved caspase 3 staining showed a higher number of apoptotic cells in spheroids when treated with EP4 antagonist, whereas the number of Ki67-positive proliferating cells were not altered (Fig. 3D,E). Consistent with these results, spheroids that were generated from *EP4* cKO mouse colonic epithelia showed significantly impaired growth compared to those obtained from control *Villin-cre* mice (Fig. 3F,G). Taken together, our results clearly indicated that epithelial EP4 is required for cell survival and the maintenance of colonic epithelial cell homeostasis.

### Microarray analyses indicated that deletion of epithelial EP4 enhances immune responses

**Figure 4:**
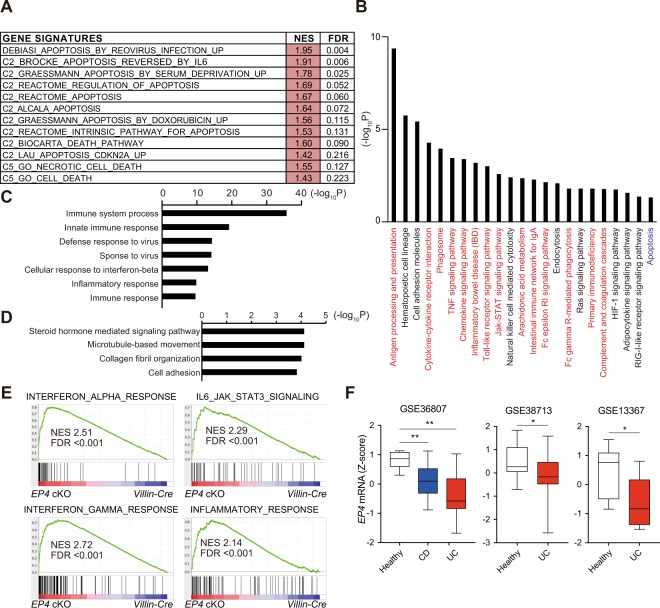
Gene expression profiling revealed the potential inflammatory status of *EP4* cKO colons. (**A**) GSEA of transcriptomic data from microarray on *EP4* cKO versus *Villin-Cre* colons. (**B**) KEGG pathways positively enriched in *EP4* cKO mouse colons analyzed with DAVID. (**C**) Biological processes (GO) positively enriched in *EP4* cKO mouse colons analyzed with DAVID. (**D**) Biological processes (GO) negatively enriched in *EP4* cKO mouse colons analyzed with DAVID. (**E**) GSEA plots of enrichment of the indicated gene signatures in *EP4* cKO versus *Villin-Cre* colons using the “Hallmarks” compilation from Molecular Signature Database (MSigDB, Broad Institute). NES, normalized enrichment score; FDR, false discovery rate. (**F**) *EP4* mRNA levels in the indicated IBD datasets. Box and whiskers graphs indicate the median and the 25th and 75th percentiles, with minimum and maximum values at the extremes of the whiskers. *P < 0.05, **P < 0.01.

To thoroughly understand the phenotype of *EP4* cKO mouse colon under physiological conditions, we performed cDNA microarray analyses to compare the gene expression profiles between control *Villin-Cre* and *EP4* cKO colon tissues. Consistent with the increased apoptosis observed in *EP4* cKO epithelia, gene-set enrichment analysis (GSEA) revealed a significant enrichment of genes involved in apoptosis-related pathways in the *EP4* cKO colons (Fig. 4A). Furthermore, results of KEGG pathway analysis using the Database for Annotation, Visualization and Integrated Discovery (DAVID) functional annotation tool showed significant enrichment of the “apoptosis” pathway in the genes dysregulated by *EP4* deletion (Fig. 4B). However, more importantly, results of KEGG pathway analysis demonstrated that inflammation-associated pathways were highly enriched and revealed more than half of the top 20 pathways related to immune response (Fig. 4B). These findings suggested that the defects in mucosal barrier function caused by elevated epithelial apoptosis and decreased secretory cell lineages led to enhanced inflammatory activity in the EP4-deficient colonic mucosa. In support with this notion, results of gene ontology (GO) analyses suggested that biological processes, such as immune system process, innate immune response, defense response to virus, inflammatory response, and immune response, were enriched in *EP4* cKO mouse colons (Fig. 4C). On the other hand, microtubule-based movement, collagen fibril organization, and cell adhesion were suppressed in *EP4* cKO mouse colons (Fig. 4D). In addition, results of GSEA showed the positive enrichment of gene signatures associated with inflammatory pathways and immune reactions in *EP4* cKO mice (Fig. 4E). These pathway analyses were highly consistent with the observed skewed crypt architecture and suggested the potent inflammatory phenotype in the *EP4* cKO colon.

To evaluate the relevance of our findings to human patients, we compared the genes that were altered by EP4 depletion to a 92 IBD-associated gene panel that was recently reported^15^. Interestingly, a total of 29 genes that showed altered expression patterns in EP4-deficient colons (log2 fold change >1) was included in the IBD-associated gene panel (Supplementary Table S2). These overlapping genes encoded the molecules such as cytokines (*Il1b, Il21, Il33*, and *Tnf*), chemokines (*Ccl4, Ccl2, Cxcl9*, and *Cxcl10*), immune cell surface or derived molecules (*Cd4, Cd86, S100a8, S100a9*, and *Nos2*), bacterial sensing-associated genes (*Nod2* and *Lcn2*), intracellular signaling molecules (*Gata3* and *Stat1*), components of the complement system (*C3* and *Cd55*), and adhesion factor (*Icam1*). Consistent with the above findings, human IBD dataset analyses demonstrated significant downregulation of *EP4* mRNA expression levels in both Crohn’s disease and ulcerative colitis patient samples relative to those in the healthy controls (Fig. 4F). Taken together, EP4-deficient colons harbored genetic alterations associated with increased apoptosis and enhanced inflammatory phenotype.

### Deletion of epithelial EP4 increases immune cell infiltration in colonic stroma

**Figure 5:**
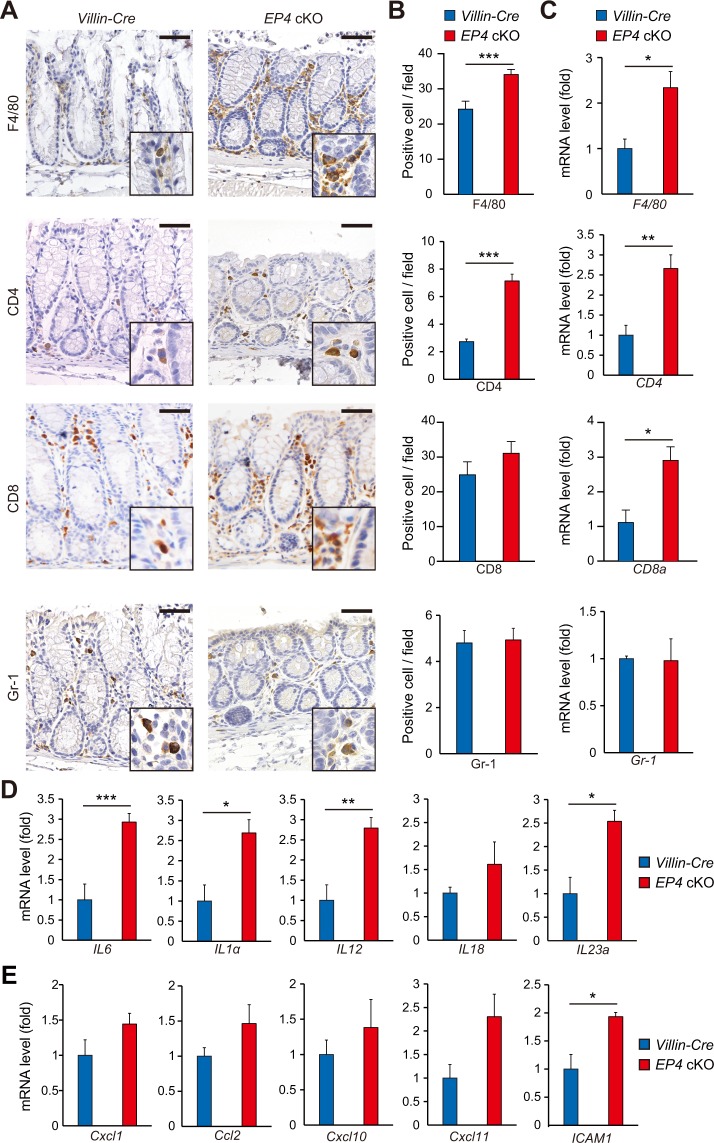
Immune cell infiltration in *EP4* cKO colons. (**A**,**B**) Staining for F4/80, CD4, CD8, and Gr-1 (**A**) in colons from *Villin-Cre* and *EP4* cKO mice, and quantification (**B**; n = 3). Scale bars = 50 μm. (**C**) mRNA expression levels of indicated genes in *Villin-Cre* and *EP4* cKO colons analyzed by qRT-PCR (n = 4). (**D**) mRNA expression levels of inflammatory cytokines in *Villin-Cre* and *EP4* cKO colons analyzed by qRT-PCR (n = 4). (**E**) mRNA expression levels of chemokines in *Villin-Cre* and *EP4* cKO colons analyzed by qRT-PCR (n = 4). Results are shown as mean ± SEM. *P < 0.05, **P < 0.01, ***P < 0.005.

To better characterize the inflammatory responses in the *EP4* cKO colon, we first examined immune cell infiltrations by immunohistochemical analysis. The numbers of F4/80^+^ macrophages and CD4^+^ T cells were higher in *EP4* cKO mouse colons, whereas numbers of CD8^+^ T cells and Gr-1^+^ neutrophils were not significantly different from those of control *Villin-Cre* mice (Fig. 5A,B). Furthermore, results of qRT-PCR analyses also showed upregulated *F4/80*, *CD4* and *CD8a* mRNA expression levels in *EP4*-deficient colons (Fig. 5C). These data suggested that deletion of EP4 in colonic epithelia affects the stroma and leads to potential inflammatory status. Consistent with this notion, mRNA expression levels of inflammatory cytokines and chemokines were elevated by the deletion of EP4 in the colonic epithelium (Fig. 5D,E).

### Epithelial EP4 deficiency exacerbates DSS-induced colitis

**Figure 6:**
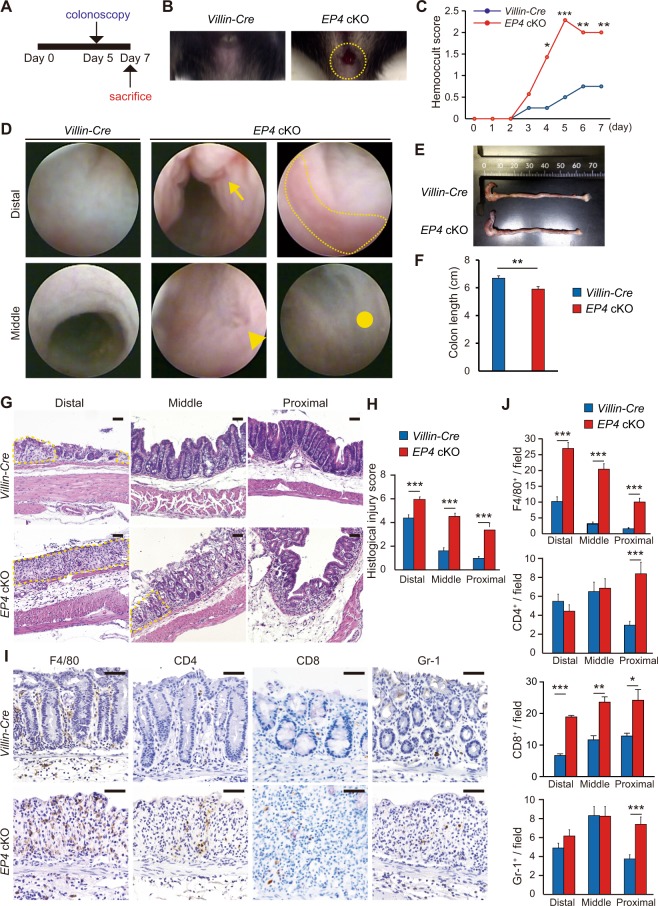
Inflammation was severely exacerbated in *EP4* cKO mice in DSS-colitis model. (**A**) Strategy of experimental colitis with 2% DSS treatment. (**B**) Representative macroscopic views of the mouse anus at day 5 after starting DSS treatment. Yellow dashed circle denotes the active bleeding of *EP4* cKO mice. (**C**) Hemooccult score at indicated time points in *Villin-Cre* (n = 8) and *EP4* cKO mice (n = 7). (**D**) Colonoscopic images of distal or middle colons from *Villin-Cre* and *EP4* cKO mice. Arrow denotes longitudinal erosion, arrowhead denotes ulcer, dotted line outlines inflammatory mucosa, and circle marks fibrotic region. (**E**,**F**) Representative macroscopic image (**E**) and length (**F**) of the colon from *Villin-Cre* and *EP4* cKO mice at day 7 after starting DSS treatment. (n: *Villin-Cre* = 8 and *EP4* cKO mice = 7). (**G**) H&E staining of colons from *Villin-Cre* and *EP4* cKO mice at day 7 after starting DSS treatment. Dotted line outlines ulcerations. Scale bars = 50 μm. (**H**) Histlogical injury scores in *Villin-Cre* (n = 8) and *EP4* cKO mice (n = 7) at day 7 after starting DSS treatment. (**I**,**J**) Staining (**I**) for F4/80, CD4, CD8, and Gr-1 of colons in *Villin-Cre* and *EP4* cKO mice at day 7 after starting DSS treatment, and quantification (**J**). Scale bars = 50 μm. (n: *Villin-Cre* = 8 and *EP4* cKO mice = 7). Results are shown as mean ± SEM. *P < 0.05, **P < 0.01, ***P < 0.005.

Given the potential inflammatory status of the *EP4* cKO colon, we next examined a DSS-induced colitis model. To induce colitis, mice were administered with 2% DSS for 7 days and subsequently analyzed by colonoscopy, followed by macroscopic and histological analyses (Fig. 6A). *EP4* cKO mice developed bloody stools after 5 days of DSS treatment (Fig. 6B). Thereafter, most DSS-treated *EP4* cKO mice presented gross bleeding, whereas hemoccult was the most severe condition observed in control *Villin-Cre* mice treated with DSS (Fig. 6C). Results of mouse colonoscopy demonstrated multiple erosions or ulcers in DSS-treated *EP4* cKO mouse colons (Fig. 6D). On day 7, macroscopic examination of the dissected colons showed that colon lengths were significantly shorter in *EP4* cKO mice (Fig. 6E,F). Histologically, *EP4* cKO mouse colons displayed more severe epithelial destruction and extensive intestinal ulceration relative to control *Villin-Cre* mouse colons (Fig. 6G). Furthermore, histological injury scores were higher in all parts of *EP4* cKO colons (Fig. 6H). Infiltration with inflammatory cells, especially F4/80-positive macrophages and CD8-positive T cells, was more prominent in *EP4* cKO mice (Fig. 6I,J). Thus, in addition to the potential inflammatory status under physiological conditions, *EP4* cKO mice were susceptible to DSS administration and developed severe colitis.

## Discussion

Our current findings demonstrated that intestinal epithelium-specific EP4 deletion led to a skewed crypt architecture and increased apoptosis of colonic epithelial cells, leading to a potential inflammatory phenotype that is associated with higher susceptibility to experimental colitis. These results shed light on the pivotal function of epithelial EP4 in maintaining colonic homeostasis and in suppressing the development of inflammation.

We observed no gross alterations in the appearance of intestines between control *Villin-Cre* and *EP4* cKO mice; however, the depth of crypts in the distal colon was significantly lower in *EP4* cKO mice. The intestinal epithelium is characterized by its rapid cell turnover, and equilibrium is maintained between proliferation of crypt cells and apoptosis of surface epithelial cells. In the present study, the deletion of epithelial EP4 did not affect cell proliferation in the colonic crypt. Consistent with this, factors that regulate crypt cell proliferation, such as β-catenin, c-Myc, and cyclin D1, showed no significant changes in mRNA expression levels or protein localization. These findings also keep in line with that there was no significant alteration (although slight decrease was observed in the case of *Lgr5*) in the expressions of ISC markers such as *Lgr5* and *Sox9* between EP4-deficient and -sufficient colons. However, since there is a gradient in EP4 expression in intestinal epithelium with more enrichment in surface tip than crypt bottom as we and others showed^1,11^, the loss of epithelial EP4 could affect more on upper part of crypt than the stem cell zone. Given that *Villin-cre* mouse line is an adequate model only to investigate epithelial cell biology, further studies with the selective inactivation of EP4 in stem or progenitor cell population using an ISC-specific Cre mouse targeting such as *Lgr5* and *Sox9* would be necessary to robustly understand the role of EP4 in ISC functions. While proliferation was not changed, the number of apoptotic cells was significantly higher in the surface epithelial cells of EP4-deficient colons. This could be explained by alterations in the mitochondrial cytochrome c-mediated cell apoptosis pathway induced by the deletion of epithelial EP4 and suggested that EP4 is required to suppress this pathway, although the detailed mechanisms by which EP4 regulates this pathway remain to be explored. Furthermore, our findings suggested that increased cell apoptosis can lead to crypt shortening, even with preserved cell proliferation potential in *EP4* cKO mice. This notion is supported by our spheroid experiments showing that treatment with an EP4 antagonist did not affect spheroid proliferation but increased the rate of apoptosis, resulting in impaired spheroid growth.

The other finding of our *in vivo* and *ex vivo* models is that epithelial-EP4 loss leads to the reduction in the numbers of secretory cell lineages including goblet, tuft, and enteroendocrine cells. Secretory cells are central to control intestinal biology, and their dysfunction leads to the pathological conditions such as inflammation. Mucus, secreted by goblet cells, forms layers that separates the bulk of the luminal contents from the intestinal epithelium, whereas intestinal tuft cells regulate a type-2 immune reaction in a response to IL13 secreted by ILC2s^16^. Consistent with our results, recent report demonstrated the importance of PGE_2_/EP4 axis in mucin secretion from goblet cells in response to IL13^17^. Mechanistically, binding of PGE_2_ to EP4 receptors stimulates cAMP-dependent exocytosis in the colon. Moreover, PGE_2_ induced cAMP response element-binding protein/ATF1 phosphorylation, which further activates Muc2 transcription in colon epithelial cells^18^. On the other hand, PGE_2_/Cox2/Dclk1 axis is shown to be an important mediator of tuft cell function under bacterial-induced colitis by enhancing epithelial repair responses^19^. We here proposed the new underlying mechanism whereby PGE_2_/EP4 regulates secretory cell proportions possibly through inhibiting cell apoptosis. Given the crucial functions of secretory cell lineages and PGE_2_/EP4 signaling pathway to suppress colitis, our study has provided with an impactful evidence that links these two important regulators in control of the development of intestinal inflammation.

In addition to increased epithelial cell apoptosis, microarray analysis demonstrated that the deletion of epithelial EP4 enhanced immune and inflammatory responses. In agreement with the defect of mucosal barrier functions in EP4-deficient colons due to increased epithelial apoptosis and reduced secretory lineages, we observed greater immune cell infiltration in colonic stroma accompanied by upregulated expression of inflammatory cytokines in *EP4* cKO mice even though the mice were comparable to WT mice in gross appearance with normal gaining weight and life span under basal conditions. These findings suggest that epithelial EP4-deficient colons are predisposed to inflammatory phenotype. This latent inflammation of the mice with epithelial EP4-deficiency is manifested by the treatment with inflammatory stimuli such as DSS. Our observations are consistent with previous studies demonstrating that global *EP4* KO mice showed downregulated expression of genes associated with tissue defense, remodeling, and immunosuppression, and upregulated expression of genes induced by IFN-γ, and revealed severer inflammation when treated with DSS^9^. Thus, we for the first time demonstrated that intestinal epithelium deficient for EP4 is already in pre-inflammatory condition and epithelial EP4 loss alone is sufficient to induce this phenotype in mouse colons. Although further studies are required to determine the role of stromal EP4 in the intestines, it is highly likely that the preparatory status for inflammatory stimuli is associated with increased epithelial apoptosis in *EP4* cKO mice. Namely, epithelial cell apoptosis induced by EP4-deficiency, which causes defects in the intestinal barrier, might precede potential and imperceptible inflammatory status in the microenvironment. If so, it is also reasonable that *EP4* cKO mice are highly susceptible to DSS treatment. Previous studies indicated that global knockout of EP4 or administration with EP4 antagonists exacerbates DSS-induced colitis^9^. In those experiments, EP4 receptors were deleted or inhibited in both epithelial cells and stromal cells. In epithelial cells, activation of EP4 signaling promotes mucin and bicarbonate secretion from epithelial cells^13,14^. In stromal cells, activation of EP4 signaling suppresses cell proliferation and Th1 cytokine production of isolated lamina propria mononuclear cells^9^. In fact, EP receptors are expressed in naive CD4^+^ T cells^3^, and EP4 is one of the most abundant and potent EP receptors expressed in Th17 cells^2^. Our current findings indicated that EP4 is required for epithelial cell survival and that EP4 deletion in epithelial cells is involved in the initiation and/or maintenance of intestinal inflammation. These findings, together with the results of our bioinformatic analyses on human IBD datasets, may contribute to a better understanding of how EP4 exerts a protective effect in human IBD^10,20^.

In conclusion, our study is the first to demonstrate the latent and potential inflammatory status in the colons induced by epithelial-specific EP4 deletion under physiological conditions. Notably, disruption of epithelial EP4 signaling is sufficient to produce such a phenotype. These data provide us with a clue to understand the mechanisms underlying the maintenance of colon homeostasis.

## Materials and Methods

### Mice

*Villin-Cre* mice were obtained from Jackson Laboratories (#004586)^21^, and *EP4 flox* mice were generated as previously described^12^. Animals were housed under specific pathogen-free conditions at the Animal Facilities of Kyoto University. Eight-week-old mice were used. All experiments were approved by the animal research committee of Kyoto University and performed in accordance with Japanese government regulations.

### Histological analyses

Mouse colonic tissues were fixed with 4% buffered paraformaldehyde solution, paraffin-embedded, and sectioned (5 µm in thickness). Sections were deparaffinized, rehydrated, and stained with hematoxylin and eosin (H&E) or alcian blue. For immunohistochemistry, sections were incubated with primary antibodies for 2 h at room temperature, then with biotinylated secondary antibody for 1 h at room temperature; immunoperoxidase labeling was visualized with avidin/biotin complex (VECTASTAIN Elite ABC kit; Vector Laboratories, Burlingame, CA, USA), and sections were colored with diaminobenzidine substrate (Dako, Santa Clara, CA, USA) and counterstained with hematoxylin. For immunofluorescence, sections were incubated with primary antibodies for 2 h at room temperature and washed with PBS. Washed sections were incubated with fluorescent dye-conjugated secondary antibody (Invitrogen, Carlsbad, CA, USA) for 1 h at room temperature, stained with Hoechst dye for 10 min at room temperature, and embedded with 50% glycerol. For quantitative analyses, cell numbers were counted in at least five random high-power fields from three animals for each genotype. Primary antibodies used in this study were obtained from the indicated suppliers: rabbit anti-Dclk1 (ab31704, Abcam), rabbit anti-Chromogranin A (ab15160, Abcam), rat anti-Ki67 (652402, BioLegend), rabbit anti-Muc2 (SC15334, Santa Cruz), rabbit anti-cleaved Caspase 3 (9664S, Cell Signaling Technology), mouse anti-E-cadherin (610182, BD Transduction Laboratories), rabbit anti-F4/80 (ab6640, Abcam), rat anti-CD4 (14-0041-85, eBioscience), rat anti-Gr1 (14-5931-85, eBioscience), mouse anti-Ctnnb1 (610153 BD Biosciences), rat anti-CD8 (550281, eBioscience), and rabbit anti-EP4 (ab133170, Abcam). TUNEL staining was performed using *In Situ* Cell Death Detection Kit, TMR red (Roche).

### Colitis model and histological injury score

To analyze the severity of colitis, mice were sacrificed after seven-days administration of 2% DSS. The colon was divided into the proximal, middle, and distal portions. Histological injury was scored as described previously^20^. Two parameters were measured: the extent of inflammation (0, none; 1, slight; 2, moderate; 3, severe) and the extent of crypt damage (0, none; 1, the basal one-third portion damaged; 2, the basal two-thirds portion damaged; 3, the entire crypt damaged but the surface epithelium intact; 4, the entire crypt and epithelium lost). Each score was added to determine the severity of colitis.

### Colon crypt isolation

Colonic crypts were isolated according to the previous protocols^22^. In brief, excised colonic tissues were incubated in TrypLE™ Express Enzyme (1x) and phenol red (#12605036) for 30 min at 37 °C. After the incubation, epithelium was separated by vigorous shaking, and the remaining intestinal tissue was placed in a new tube for collection of subsequent fractions. After the isolation, crypt cells were pelleted, and passed through 100 µm cell strainers. Then, colonic crypts were collected for either spheroid culture or RNA analyses.

### Spheroid culture

Conditioned medium of L-cell line secreting Wnt3a, R-spondin3, and Noggin (L-WRN CM) was prepared as described previously^23^. L-WRN cells were purchased from ATCC (ATCC; CRL3276). Isolated crypts of the proximal colon were embedded in Matrigel (BD Biosciences). For spheroid culture of normal epithelium, 50% L-WRN CM supplemented with 5 µM Y-27632 (Tocris Bioscience) was added to each well. For EP4 antagonist experiment, spheroids generated from control (*Villin-Cre*) mice were treated with or without specific EP4-antagonist L161,982 (100 µM, Sigma) for 48 h and then analyzed.

### RNA extraction and analysis

Total RNA was subsequently extracted using the RNA mini Kit (Qiagen, Hilden, Germany). RNA was either processed for microarray or quantitative reverse-transcription polymerase chain reaction (qRT-PCR). Single-strand complementary DNA (cDNA) was synthesized using a Transcriptor First Strand cDNA Synthesis Kit (Roche Applied Science, Basel, Switzerland). qRT-PCR was performed using SYBR Green I Master (Roche Applied Science) and a Light Cycler 480 (Roche Applied Science). All reactions were performed in duplicate, and expression levels of mRNAs were normalized comparing to those of glyceraldehyde 3-phosphate dehydrogenase (GAPDH) mRNA. Primers were designed using the MGH primer bank, and primer sequences are described in Supplementary Table S1.

### Microarray analysis

Total RNAs from *Villin-Cre* and *Villin-Cre; EP4*^*flox/flox*^ mouse whole colon tissues (n = 3, respectively) were subjected to the SurePrint G3 Mouse GE v2 8 × 60 K Microarray (Agilent). Processed signal intensities were normalized by the global scaling method. A trimmed mean probe intensity was determined by removing 2% of the lower and the higher end of the probe intensities to calculate the scaling factor. Normalized signal intensities were then calculated from the target intensity on each array using this factor, such that the trimmed mean target intensity of each array was arbitrarily set to 2500. Data were analyzed to identify gene probes that showed more than a two-fold change. The accession number for the complete microarray data reported in this paper is GEO: GSE 135859.

### Bioinformatic analysis

KEGG and GO analyses on mouse microarray data (see above “Microarray analysis”) was performed by uploading the data to DAVID. Gene Set Enrichment Analysis (GSEA) was performed using GSEA 3.0 software (http://www.broadinstitute.org/gsea/index.jsp) with 1000 gene-set permutations using the signal-to-noise gene-ranking metric with the collections h.all.v6.1.symbols (H), c2.all.v6.1.symbols (C2), or c5.all.v6.1.symbols (C5). Raw gene expression data inflammatory bowel disease sample datasets (GSE36807, GSE38713, and GSE13367) were directly accessed through the GEO (https://www.ncbi.nlm.nih.gov/geo/).

### Electron microscope

The mouse colon tissues were placed in 4% paraformaldehyde with 2% glutaraldehyde for a minimum of 12 h, and cut into 1 mm thick coronal sections. Next, sections were post-fixed in 1.0% osmium tetraoxide in 100 mM phosphate buffer, pH 7.4, for 2 h at room temperature, and dehydrated in a series of graded ethanol solutions. After immersion in propylene oxide, samples were immersed in a mixture (1:1) of propylene oxide and Epon812 (LUVEAC-812, Nacalai Tesque, Japan) for 1.5 h. Samples were then immersed in a mixture (1:3) of propylene oxide and Epon812 for 1.5 h and finally immersed in only Epon812 for 12 h. After immersion, samples were embedded in Epon812 resin according to the inverted beam capsule procedure and polymerized at 60 °C for 3 days. The tissue samples were cut into ultrathin sections (70 nm) on an EM UC6 ultramicrotome (Leica, Germany). The ultrathin sections were examined with an H7650 electron microscope (Hitachi, Japan).

### Colonoscopy procedures

To analyze the severity of colitis in the mouse colon, we used a high-resolution miniaturized endoscopic system^24–26^. This system is composed of a miniature rigid endoscope, a xenon light source, a triple chip high resolution CCD camera, and an operating sheath with 3 Fr. instrument channel and water injection bulb to regulate inflation of the mouse colon (all from Karl Storz). Endoscopic images were viewed with high resolution on a flat panel color monitor.

### Statistical analysis

All values are presented as mean ± SEM. Significant differences between groups were determined using a Student’s t test (two-tailed unpaired) when the data met the normal distribution tested by D’Agostino test. If the data did not meet this test, a Mann-Whitney test was used. The significance level for statistical testing was set at *P* < 0.05.

## Supplementary information

Supplementary Information
