# Supplementary material for: Epithelial EP4 plays an essential role in maintaining homeostasis in colon
Source: Sci Rep. 2019 Oct 23;9:15244. doi: 10.1038/s41598-019-51639-2 (PMC6811535; doi:10.1038/s41598-019-51639-2)
Supplement: Supplementary file 1 — Supplementary Information [file 41598_2019_51639_MOESM1_ESM.pdf]

Supplementary Information

Epithelial EP4 plays an essential role in maintaining homeostasis in colon

Running title: Loss of epithelial EP4 drives inflammatory phenotype in colon

Yoshihide Matsumoto, Yuki Nakanishi, Takuto Yoshioka, Yuichi Yamaga, Tomonori Masuda, Yuichi Fukunaga, Makoto Sono, Takaaki Yoshikawa, Munemasa Nagao, Osamu Araki, Satoshi Ogawa, Norihiro Goto, Yukiko Hiramatsu, Richard M. Breyer, Akihisa Fukuda, Hiroshi Seno

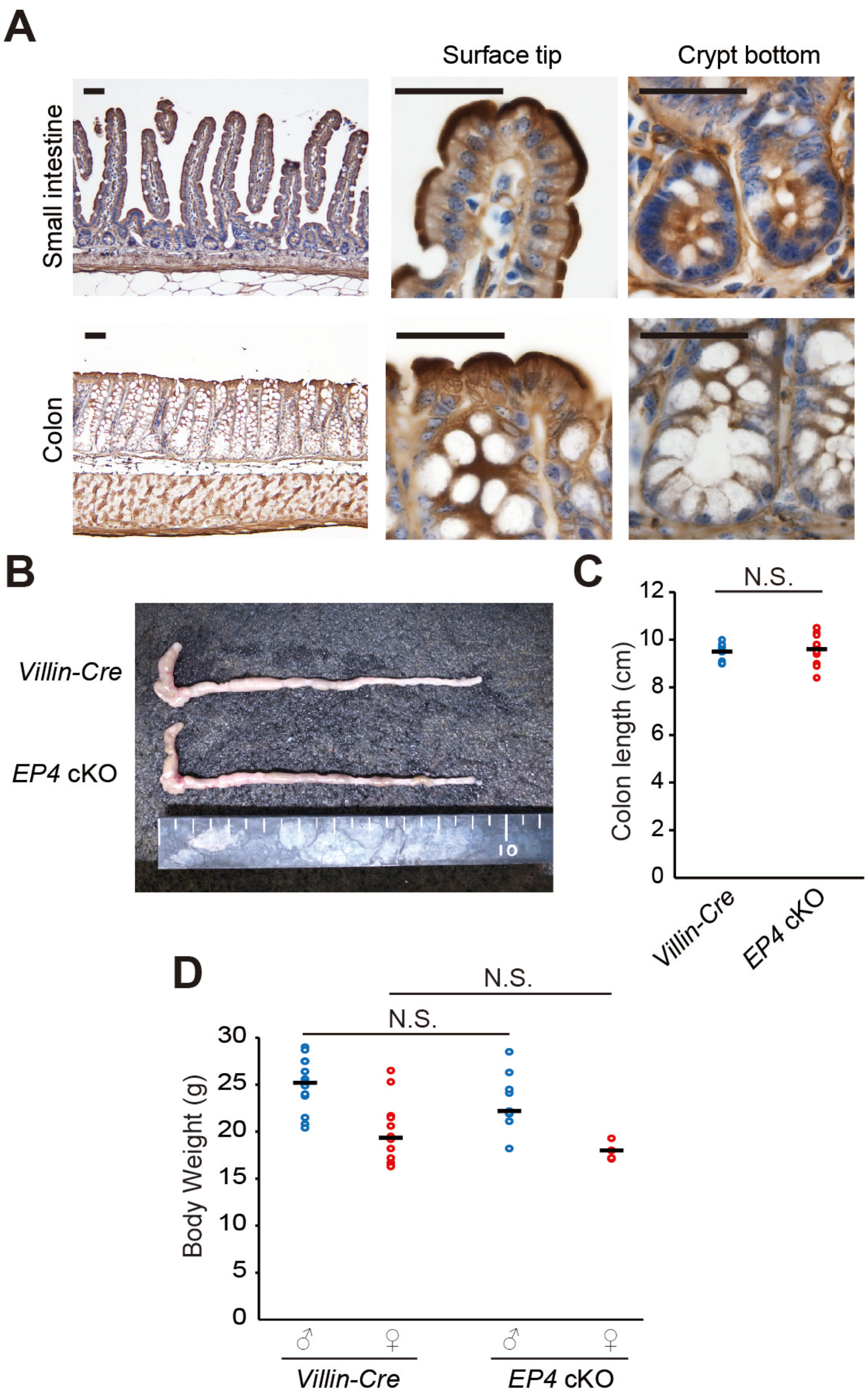

Figure S1. *EP4* cKO mice did not show significant alteration macroscopically. (A) EP4 staining of *Villin-Cre* mouse colons. Scale bars=50 μm. (B) Representative macroscopic view of the colon of *Villin-Cre* and *EP4* cKO mice. (C) Colon length of *Villin-Cre* (n = 14) and *EP4* cKO mice (n = 17). (D) Body weight of *Villin-Cre* (n: male = 14, female = 12) and *EP4* cKO mice (n: male = 9, female = 5). N.S.: not significant.

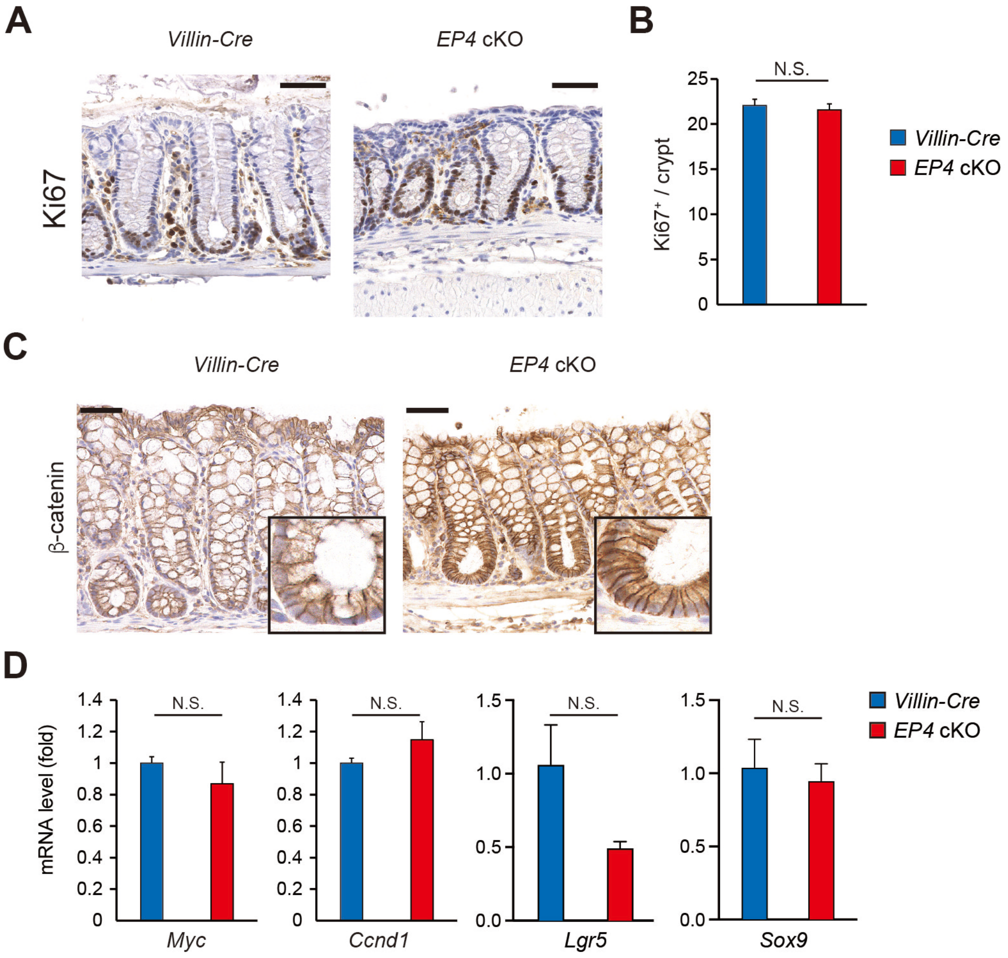

Figure S2. Proliferative status was not changed in *EP4* cKO mice. (A) Ki67 staining of *Villin-Cre* and *EP4* cKO mouse colons. Scale bars=50 μm. (B) Quantification of Ki67$^+$ cells to crypt in *Villin-Cre* and *EP4* cKO colons (n = 3). (C) β-catenin staining of *Villin-Cre* and *EP4* cKO mice. Scale bars=50 μm. (D) mRNA expression levels of indicated genes in *Villin-Cre* and *EP4* cKO mice (n = 3 or 4) analyzed by qRT-PCR. Results are shown as mean ± SEM. N.S.: not significant.

Table S1: Primer sequence used for qRT-PCR analysis

EP4	CCATTCCCGCAGTGATGTTCA	TGCGCGACTTGCACAATACTA
Muc2	TCCAGGTCTCGACATTAGCAG	GTGCTGAGAGTTTGCGTGTCT
ChromoglaninA	CAGGGACACTATGGAGAAGAGA	GGTGATTGGGTATTGGTGGCT
Dclk1	AGGGTGGAGACCTTTTCGAT	CAGGCTGTGCAGGTATTTGA
Bak	CAGCTTGCTCTCATCGGAGAT	GGTGAAGAGTTCGTAGGCATTC
Bid	GCCGAGCACATCACAGACC	TGGCAATGTTGTGGATGATTTCT
Bim	TCGTCCATCGAGGATGACTTC	TGCAGAGAGAGGATACTGTAGAC
Bax	TGAAGACAGGGGCCTTTTTG	AATTCGCCGGAGACACTCG
Bad	TGAGCCGAGTGAGCAGGAA	GCCTCCATGATGACTGTTGGT
Noxa	CGAGCCCTGGCCTATATGATA	GGGTCCTTTGTAGCTGCTCC
Bcl2	GGGAGAACAGGGTATGATAACCG	TAGCCCCTCTGTGACAGCTTA
F4/80	TGACTCACCTTGTGGTCCTAA	CTTCCCAGAATCCAGTCTTTCC
CD4	AGGTGATGGGACCTACCTCTC	GGGGCCACCACTTGAACTAC
CD8a	CCGTTGACCCGCTTTCTGT	CGGCGTCCATTTTCTTTGGAA
Gr-1	GACTTCCTGCAACACAACTACC	ACAGCATTACCAGTGATCTCAGT
IL6	TAGTCCTTCCTACCCCAATTTCC	TTGGTCCTTAGCCACTCCTTC
IL1α	CTGATGAAGCTCGTCAGGCAG	TGGTGCTGAGATAGTGTTTGTC
IL12	ACTCTGCGCCAGAAACCTC	CACCCTGTTGATGGTCACGAC
IL18	GACTCTTGCGTCAACTTCAAGG	CAGGCTGTCTTTTGTCAACGA
IL23α	AGCAACTTCACACCTCCCTAC	ACTGCTGACTAGAACTCAGGC
Cxcl1	CTGGGATTCACCTCAAGAACATC	CAGGGTCAAGGCAAGCCTC
Ccl2	TTAAAAACCTGGATCGGAACCAA	GCATTAGCTTCAGATTTACGGGT
Cxcl10	CCAAGTGCTGCCGTCATTTTC	GGCTCGCAGGGATGATTTCAA
Cxcl11	TGTAATTTACCCGAGTAACGGC	CACCTTTGTCGTTTATGAGCCTT
ICAM1	GTGATGCTCAGGTATCCATCCA	CACAGTTCTCAAAGCACAGCG
Myc	TCTCCATCCTATGTTGCGGTC	TCCAAGTAACTCGGTCATCATCT
Ccnd1	CAGAAGTGCGAAGAGGAGGTC	TCATCTTAGAGGCCACGAACAT
Lgr5	TCCTAGAAGAGTTACGTCTTGCT	CCTTGGGAATGTGTGTCAAAGC
Sox9	AGTACCCGCATCTGCACAAC	ACGAAGGGTCTCTTCTCGCT

Table S2: Twenty nine common genes between 92 IBD-associated gene panel [21]and altered genes in EP4-deficient colon (Log2-scale >1)

Il21
Ptpn22
Gata3
Klrk1
Cxcr3
Nod2
Ccl4
Cd4
Cd86
Tnf
Cd44
Col1a2
Ltf
Il33
Ccl2
Vwf
Isg15
Stat1
Icam1
C3
S100a8
Mmp7
Cxcl10
Cxcl9
Cd55
S100a9
Il1b
Nos2
Lcn2
